# Aerobic fitness in children with cerebral palsy compared to typically developing peers: A systematic review and meta-analysis

**DOI:** 10.1016/j.bjpt.2024.101142

**Published:** 2024-11-15

**Authors:** Emma J. Wijnhoud, Arnoud M.M. Edelman Bos, Annemieke I. Buizer, Heleen Beckerman

**Affiliations:** a Department of Rehabilitation Medicine, Amsterdam UMC, location VU University, Amsterdam, the Netherlands; b Faculty of Medicine, Vrije Universiteit Amsterdam, Amsterdam, the Netherlands; c Amsterdam Movement Sciences Research Institute, Rehabilitation and Development, Amsterdam, the Netherlands; d Emma Children's Hospital, Amsterdam, the Netherlands; e Amsterdam Public Health research institute, Societal Participation and Health, Amsterdam, the Netherlands

**Keywords:** Aerobic capacity, Cardiorespiratory fitness, Exercise testing, Maximal oxygen consumption, VO_2peak_

## Abstract

**Background:**

In the public health domain, aerobic fitness is an important predictor of both health and disease.

**Objective:**

To determine aerobic fitness in children with cerebral palsy (CP) compared to typically developing (TD) peers measured with a maximal exercise test.

**Methods:**

A systematic literature search was conducted in PubMed (MEDLINE), PsycArticles, PsycInfo, CINAHL, and SPORTDiscus (EBSCO). Original studies that reported findings on aerobic fitness expressed as peak oxygen uptake (VO_2peak_) during a maximal exercise test measured with a gas analysis system, in children with CP, aged 18 years or younger, were included. VO_2peak_ values were pooled, using the generic inverse variance method, for type of maximal exercise test, Gross Motor Function Classification System (GMFCS) level, distribution of CP, and sex.

**Results:**

Thirty-six studies with a total of 510 children with CP (GMFCS I-IV) and 173 TD peers were included. VO_2peak_ was measured using cycle ergometer test (*n* = 16), treadmill exercise test (*n* = 13), arm crank ergometer test (*n* = 6), shuttle run test (*n* = 3), and shuttle ride test (*n* = 1). The overall pooled VO_2peak_ in children with CP was 32.84 mL/kg/min (SE 1.28) and 45.02 mL/kg/min (SE 1.32) in TD peers, with a difference between CP and TD of -12.17 mL/kg/min (95 % CI: -16.70, -7.64). Subgroup analyses revealed that aerobic fitness was most compromised in children at higher GMFCS levels and boys with CP.

**Conclusion:**

Aerobic fitness is severely compromised in children with CP. Promoting a healthy lifestyle and increasing participation in physical activities for young people with CP is recommended.

The study protocol was prospectively registered in the PROSPERO registry with reference number CRD42021292879.

## Highlights

• Children with cerebral palsy reach lower VO_2peak_ values compared with typically developing peers.
• Low aerobic fitness has immediate and long-term negative health consequences.
• Promoting a healthy lifestyle and increase of habitual physical activities is warranted.
• Intersectoral teamwork is required to overcome barriers to being physically active.

## Introduction

Cerebral palsy (CP) is the most common motor disability in childhood. CP is an umbrella term for a group of permanent disorders that are attributed to non-progressive disturbances that occurred in the developing fetal or infant brain. CP is diagnosed in about 2 per 1000 live births.^1^^,^^2^ As a result of this brain abnormality, CP is characterized by persisting movement and/or posture impairments. This in turn results in many children experiencing mobility problems and limitations in physical activities.^3^ In addition to CP-related limitations in physical activity, there is also a marked general decrease in physical activity levels and increase in sedentary behavior in the current generation of children and adolescents, including children with childhood disability.^4^^,^^5^

Aerobic fitness (i.e. cardiorespiratory fitness) is an important indicator of the physical fitness of children with CP.^6^ A low aerobic fitness has clearly proven to have negative consequences for later life in young people.^4^^,^^6^, ^7^, ^8^, ^9^ For example, a strong association exists between cardiorespiratory fitness levels and cardiovascular disease risk factors.^7^, ^8^, ^9^, ^10^ Besides that, a low aerobic fitness level not only increase the risk for health problems on the longer term, but also negatively affects the performance of daily activities and societal participation in daily life.^4^^,^^5^^,^^10^ The higher the aerobic fitness reached at a young age, the greater the chances that it will be maintained during the growth period.^7^ Childhood aerobic fitness can contribute to decrease cardiovascular risk factors and diseases later in life. It is thus important to investigate the aerobic fitness in children with CP.

Cardiorespiratory fitness can be defined as the capacity of the cardiovascular and respiratory systems to deliver oxygen from the atmosphere to the skeletal muscles and use it to create energy for muscle cells to perform prolonged exercises and physical activity.^11^^,^^12^ The key parameter of aerobic fitness is the maximum oxygen uptake (VO_2max_). VO_2max_ is measured during a progressive cardiopulmonary exercise test.^13^^,^^14^ It is considered that a plateau in oxygen uptake during the final stage of the exercise test is a required criterion of attaining a true VO_2max_.^15^ Because children as well as adults do not frequently reach a VO_2_ plateau during maximal exercise testing, the peak oxygen uptake (VO_2peak_) is considered the best indicator of aerobic fitness in children.^15^

Worldwide, various studies have reported on VO_2peak_ values in children with CP compared with their typically developing (TD) peers. However, a systematic review with pooled overall VO_2peak_ values is lacking. Understanding the VO_2peak_ values in children with CP will raise awareness in children, parents, and healthcare professionals for early recognition of reduced aerobic fitness levels. Moreover, it will also serve as alert for policy makers in the field of public health. Low VO_2peak_ values in children with CP may justify the need for the facilitation and encouragement of inclusive physical activities in daily life.

Therefore, the aim of the present study was to systematically review the current literature and give an overview of the VO_2peak_ in children and adolescents with CP compared with TD peers. VO_2peak_ values were pooled per maximal exercise test, Gross Motor Function Classification System (GMFCS) level, motor distribution of CP, and sex.

## Methods

This systematic review was guided by the Preferred Reporting Items for Systematic Reviews and Meta-Analyses (PRISMA) statement.^16^ The study protocol was prospectively registered in the PROSPERO registry with reference number CRD42021292879.

### Literature search and article selection

The following electronic databases were searched: PubMed (MEDLINE), PsycArticles, PsycInfo, CINAHL, and SPORTDiscus (EBSCO). In brief, the search blocks included keywords related to: (1) VO_2peak_; (2) Children; and (3) Cerebral Palsy. The full literature search is provided in the Supplementary material Table S1.

For this review, studies using an incremental exercise test aimed at testing the maximal exercise fitness were included. Studies using sub-maximal exercise tests were excluded. Original studies that reported findings on VO_2peak_ measured with a gas analysis system, in children with CP, aged 18 years or younger, measured with a maximal exercise test were included. Exclusion criteria were (i) mixed diagnosis groups, unless data of the CP group were separately described, (ii) articles written in other languages than English, Dutch, German, or French, and (iii) systematic reviews, letters to the editor, and other studies without original VO_2peak_ data. If TD peers were tested in the included studies, their VO_2peak_ was also extracted. No exclusion criteria were applied for this control group.

Two reviewers (E.J.W. and H.B.) screened the search results by title and abstract, and subsequent full-text, using the web-tool Rayyan.^17^ Additionally, the reference lists of included studies and systematic reviews were screened for any potentially relevant studies. In case of a disagreement between reviewers, a discussion to include or exclude the article took place. The searches are up to date until November 15, 2023.

### Data extraction

A pre-designed extraction form was used to collect relevant information from each included study. Data extraction was done by one reviewer (E.J.W.) and cross-verified by a second reviewer (H.B.). Extracted study and child characteristics included: last name first author; year of publication; number of participants, mean age; sex; GMFCS level; distribution and motor type of CP; type of maximal exercise test; VO_2peak_; maximal heart rate (HR_max_); and respiratory exchange ratio (RER). When data were available, VO_2peak_ was reported per subgroup according to the type of maximal exercise test, GMFCS level, distribution type of CP, and sex. The main outcome of interest was VO_2peak_ as expressed in mL/kg/min or L/min.

The GMFCS describes the functional mobility level of children with CP.^18^^,^^19^ Children functioning at GMFCS level I are able to walk without limitations, those classified as GMFCS level II experience difficulty walking on uneven terrain, inclines, and in crowds or confined spaces. GMFCS level III reflects children who walk with a walking aid and use a wheelchair when covering longer distance. Children at GMFCS level IV are dependent on physical assistance or powered mobility in most settings, while children who are classified at GMFCS V are not able to walk or use a wheelchair by themselves.^18^^,^^19^

The motor type of CP can be subdivided into 3 types based on the dominant motor disorder: spastic, dyskinetic, or ataxic. In spastic CP, spasticity is the predominant disorder, with spasticity characterized by hypertonia and pathological reflexes, in particular increased stretch reflexes. Hypertonia can be elicited at the start of a movement, in which fast passive stretch results in a velocity dependent increase in muscle resistance. Children with spastic CP can be further characterized by the distribution of involved limbs (unilateral/bilateral). For the purpose of this study, hemiplegia was categorized as unilateral CP, whereas diplegia, tetraplegia, and quadriplegia were classified as bilateral CP. Children with dyskinetic CP predominantly have involuntary sustained or intermittent muscle contractions causing stereotyped movements and abnormal postures, which can be subdivided in dystonia and choreo-athetosis. Often, primitive reflexes persist. In ataxic CP, damage to the cerebellum causes lack of muscle coordination. Common features are balance and coordination problems.^2^

### Quality assessment of included studies

The main aim of our systematic review and meta-analysis was to summarize reported VO_2peak_ values in youth with CP in all included literature, regardless of study design or main objective of the original studies. Therefore, the most important methodological quality question for our systematic review concerned the valid, unbiased measurement of VO_2peak_. The risk of biased VO_2peak_ measurement was judged by means of three criteria: did the study report (1) how the VO_2peak_ value was defined, (2) prior stated additional peak criteria related to maximal heart rate, respiratory exchange ratio, or signs of perceived exhaustion, and (3) reporting the number of children who successfully completed the maximal exercise test.^12^^,^^15^ The results of the quality assessment are reported in Supplementary material Table S2.

### Data analysis

The study and child characteristics from the included studies were reported using descriptive statistics. The VO_2peak_ outcome of the same children was re-used in a large number of included articles. Therefore, for data pooling, unique or so-called ‘independent’ VO_2peak_ outcomes were used. Hereto, the outcomes of the article with the most complete (subgroup) data were used. In studies in which a maximal exercise test was repeatedly performed (e.g. test-retest study, intervention studies), data of the first test was used to exclude any learning and/or intervention effects. Verschuren and Takken^20^ used external reference data of 336 healthy controls for comparison. In this review, these external TD reference data were excluded. Two articles^21^^,^^22^ which fulfilled our inclusion criteria showed VO_2peak_ data only in graphs. Authors of these studies were contacted and asked to share the VO_2peak_ values needed for meta-analysis.

Meta-analyses in CP and TD subgroups were conducted on VO_2peak_ data in mL/kg/min. A limited number of studies reported VO_2peak_ in L/min or VO_2peak_ adjusted for lean body mass, resulting in insufficient data for conducting meta-analyses. We estimated the pooled VO_2peak_ with corresponding 95 % confidence intervals (CI) according to the generic inverse variance method, using a random effect model. The difference between groups was tested with an unpaired *t*-test. To assess heterogeneity between study outcomes, I^2^ statistic was used: an I^2^ value *>* 75 % was considered high heterogeneity. Meta-analysis were performed using SPSS version 28 (IBM Corp, Armonk, NY). A p-value < 0.05 was considered statistically significant.

## Results

### Search results

**Fig. 1:**
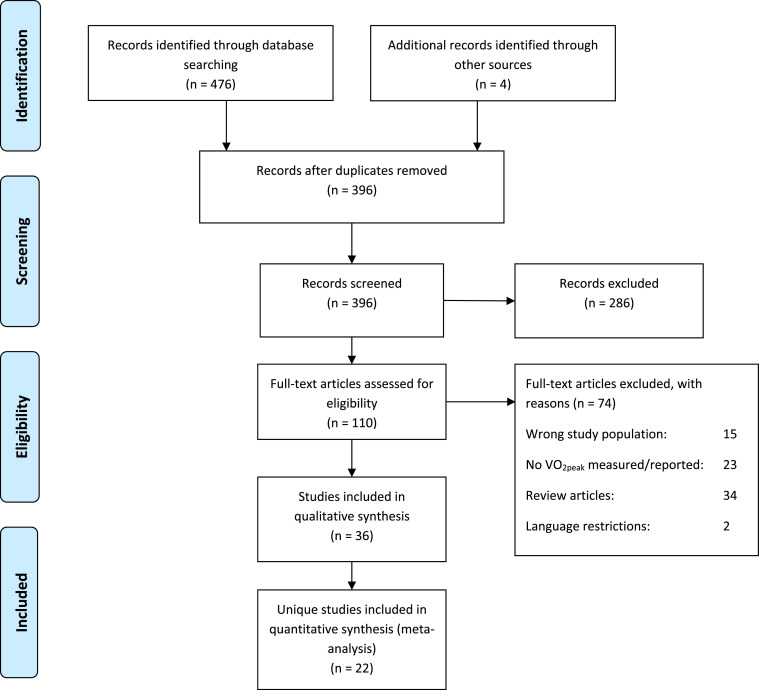
Flow diagram of study selection.

The literature search yielded a total of 480 records, of which 36 studies^20^, ^21^, ^22^, ^23^, ^24^, ^25^, ^26^, ^27^, ^28^, ^29^, ^30^, ^31^, ^32^, ^33^, ^34^, ^35^, ^36^, ^37^, ^38^, ^39^, ^40^, ^41^, ^42^, ^43^, ^44^, ^45^, ^46^, ^47^, ^48^, ^49^, ^50^, ^51^, ^52^, ^53^, ^54^, ^55^ met the inclusion criteria (Fig. 1). Children with CP were included from 13 different countries. Papers were published between 1978 and 2023. Data from 16 intervention studies, 9 cross-sectional case-control studies comparing children with CP and TD, 4 clinimetric studies (one test-retest study,^27^ and three studies^47^^,^^53^^.^^54^ comparing two different test modalities) were included. Seven studies had another study design (Table 1). Further details, including the risk of bias score of the 36 studies, ordered by the maximal exercise test used, are presented in Supplementary material Table S2.

**Table 1:** Participant characteristics of the 36 included studies. Studies are ordered alphabetically by first author and year of publication.

**Study**	**Study design**	**Included participants (*n*)**	**Sex**	**Age, *years*** **(*mean ± SD*)** **[range]**	**GMFCS level (*n*)**	**Distribution and motor type of CP (*n*)**
**Balemans et al.**^23^^,^‡	Cross-sectional case-control analysis (data from mixed designs)	TD: 31	Boys: 14 Girls: 17	10.0 ± 1.6	na	na
		CP: 70	Boys: 35 Girls: 35	9.9 ± 1.6 10.3 ± 2.4 9.5 ± 2.0	GMFCS I: 36 GMFCS II: 24 GMFCS III: 10	Unilateral spastic: 26 Bilateral spastic: 41 Dyskinetic: 2 Ataxic: 1
**Balemans et al.**^24^^,^‡	Secondary analysis RCT	CP: 46	Boys: 26 Girls: 20	9.6 ± 1.7	GMFCS I: 26 GMFCS II: 12 GMFCS III: 8	Unilateral spastic: 22 Bilateral spastic: 24
**Balemans et al.**^25^^,^‡	Secondary analysis RCT	CP: 46	Boys: 26 Girls: 20	9.6 ± 1.7	GMFCS I: 26 GMFCS II: 12 GMFCS III: 8	Unilateral spastic: 22 Bilateral spastic: 24
**Balemans et al.**^26^	Cross-sectional case-control study	TD: 20	Boys: 8 Girls: 12	11.7 ± 3.4	na	na
		CP: 37	Boys: 18 Girls: 19	11.3 ± 3.1 13.9 ± 3.6 16.3 ± 4.9	GMFCS I: 13 GMFCS II: 17 GMFCS III: 7	Unilateral spastic: 6 Bilateral spastic: 31
**Brehm et al.**^27^^,^‡	Clinimetric study (test-retest)	CP: 16	Boys: 9 Girls: 7	10.5 ± 2.1	GMFCS I: 3 GMFCS II: 11 GMFCS III: 2	Unilateral spastic: 2 Bilateral spastic: 14
**Dahlbäck and Norlin**^28^	UCT	CP: 6	NR	[9–15]	NR	Bilateral spastic: 6
**Dallmeijer and Brehm**^29^	Cross-sectional case-control study	TD: 10	Boys: 5 Girls: 5	9.8 ± 2.9	na	na
		CP: 8	Boys: 4 Girls: 4	9.9 ± 3.0	GMFCS I: 7 GMFCS II: 1	Unilateral spastic: 3 Bilateral spastic: 5
**Depiazzi et al.**^30^	Pilot RCT	CP control: 6	Boys: 2 Girls: 4	14.7 ± 2.5	GMFCS II: 6	NR
		CP intervention: 6	Boys: 3 Girls: 3	14.1 ± 1.6	GMFCS II: 6	NR
**Garcia et al.**^31^	Cross-sectional case-control study	TD: 40	Boys: 21 Girls: 19	11.0 ± 3.6	na	na
		CP: 40	Boys: 21 Girls: 19	11.0 ± 3.3	GMFCS levels I and II	Bilateral spastic: 40
**Hoofwijk et al.**^32^^,^‡	Cross-sectional case-control study	TD: 9	Boys: 7 Girls: 2	14.0 ± 2.4	na	na
		CP: 9	Boys: 7 Girls: 2	13.5 ± 2.7	NR	Unilateral spastic: 1 Bilateral spastic: 8
**Jung et al.**^33^	Preliminary case series study	TD: 2	Boys: 1 Girls: 1	9.5 ± 3.5*	na	na
		CP: 4	Boys: 3 Girls: 1	11 ± 3.4*	GMFCS I: 1 GMFCS II: 3	Unilateral spastic: 2 Bilateral spastic: 2
**Kim et al.**^34^^,^‡	Baseline data RCT	CP: 40	Boys: 21 Girls: 19	7.4 ± 1.6	GMFCS I: 21 GMFCS II: 19	Unilateral spastic: 18 Bilateral spastic: 22
**Klimek-Piskorz and Piskorz**^35^	Cross-sectional study	CP: 14	NR	[16–17]	NR	Spastic: 14
**Klimek-Piskorz et al.**^36^	Cross-sectional case-control study	TD: 10	Boys: 10 Girls:	16.1 ± 0.3	na	na
		CP: 10	Boys: 10 Girls:	16.7 ± 0.5	NR	Bilateral spastic: 10
**Klimek-Piskorz**^37^	UCT	CP: 8	Boys: 8 Girls:	17.5 ± 0.3	NR	Bilateral Spastic: 8
**Lauglo et al.**^38^	UCT	CP: 20	Boys: 11 Girls: 9	14 [13–16]#	GMFCS I: 8 GMFCS II: 4 GMFCS III: 3 GMFCS IV: 5	Unilateral spastic: 9 Bilateral spastic: 7 Dyskinetic: 3 Ataxic: 1
**Lee et al.**^39^	Baseline data RCT	CP: 39	Boys: 21 Girls: 18	7.44 ± 1.60	GMFCS I: 21 GMFCS II: 18	Unilateral spastic: 19 Bilateral spastic: 20
**Leunkeu et al.**^40^	Cross-sectional case-control study	TD: 10	NR	14 ± 0.6	na	na
		CP: 9	NR	13 ± 1.9	NR	Unilateral spastic: 4 Bilateral spastic: 5
**Leunkeu et al.**^21^	Cross-sectional case-control study	TD: 8	Boys: 8 Girls:	14 ± 1	na	na
		CP: 8	Boys: 6 Girls: 2	14 ± 1	GMFCS I: 4 GMFCS II: 4	Unilateral spastic: 4 Bilateral spastic: 4
**Nsenga Leunkeu et al.**^44^^,^‡	CCT	CP control: 12	Boys: 6 Girls: 6	14.2 ± 1.8	GMFCS I: 8 GMFCS II: 4	Unilateral spastic: 10 Bilateral spastic: 2
		CP intervention: 12	Boys: 6 Girls: 6	14.2 ± 1.9	GMFCS I: 8 GMFCS II: 4	Unilateral spastic: 10 Bilateral spastic: 2
**Nsenga et al.**^45^^,^‡	CCT	TD: 10	Boys: 6 Girls: 4	14.1 ± 2.1	na	na
		CP control: 10	Boys: 6 Girls: 4	14.2 ± 1.8	GMFCS I: 7 GMFCS II: 3	Unilateral spastic: 8 Bilateral spastic: 2
		CP intervention: 10	Boys: 6 Girls: 4	14.2 ± 1.9	GMFCS I: 7 GMFCS II: 3	Unilateral spastic: 8 Bilateral spastic: 2
**Lundberg**^41^	Cross-sectional case-control study	TD: 9	Boys: 6 Girls: 3	11.7 ± 0.5 11.7 ± 0.6	na	na
		CP: 9	Boys: 5 Girls: 4	11.4 ± 0.5 11.8 ± 0.5	NR	Bilateral spastic: 9
**Lundberg**^42^	Longitudinal study (approx. 6 years)	TD: 12	Boys: 7 Girls: 5	12.3 ± 1.2	na	na
		CP: 26	Boys: 19 Girls: 7	11.5 ± 1.9 12.0 ± 0.3 11.2 ± 2.2	NR	Unilateral spastic: 3 Bilateral spastic: 19 Dyskinetic: 4
**Maltais et al.**^43^	Cross-sectional study	CP: 11	Boys: 7 Girls: 4	13 ± 1.4	GMFCS levels I and II	Unilateral spastic: 4 Bilateral spastic: 7
**Massin and Allington**^22^	UCT	CP: 15	Boys: 9 Girls: 6	6.5 ± 2.3*	NR	Unilateral spastic: 9 Bilateral spastic: 6
**Park et al.**^46^^,^‡	Pilot RCT	CP control: 13	Boys: 8 Girls: 5	7.5 ± 1.6	GMFCS I: 7 GMFCS II: 6	NR
		CP intervention: 13	Boys: 6 Girls: 7	8.2 ± 1.9	GMFCS I: 7 GMFCS II: 6	NR
**Piskorz and Klimek-Piskorz**^47^	Clinimetric study (compares 2 test modalities)	CP: 15	Boys: 15 Girls:	[16–17]	NR	Bilateral spastic: 15
**Sansare et al.**^48^	RCT	CP control: 11	Boys: 9 Girls: 2	13.7 ± 2.9	GMFCS II: 4 GMFCS III: 4 GMFCS IV: 3	Spastic: 11
		CP intervention I: 14	Boys: 13 Girls: 1	14.5 ± 2.4	GMFCS II: 6 GMFCS III: 3 GMFCS IV: 5	Spastic: 14
		CP intervention II: 11	Boys: 8 Girls: 3	12.7 ± 2.1	GMFCS II: 2 GMFCS III: 4 GMFCS IV: 5	Spastic: 11
**Suk and Kwon**^49^^,^‡	RCT	CP control: 23	Boys: 12 Girls: 11	7.2 ± 1.5	GMFCS I: 11 GMFCS II: 10 GMFCS III: 2	Unilateral spastic: 9 Bilateral spastic: 14
		CP intervention: 23	Boys: 12 Girls: 11	7.7 ± 1.6	GMFCS I: 10 GMFCS II: 9 GMFCS III: 4	Unilateral spastic: 10 Bilateral spastic: 13
**Unnithan et al.**^50^^,^‡	Cross-sectional case-control study	TD: 9	Boys: 7 Girls: 2	13.6 ± 2.1	na	na
		CP: 9	Boys: 7 Girls: 2	12.7 ± 2.8	NR	Unilateral spastic: 1 Bilateral spastic: 8
**Unnithan et al.**^51^	CCT	CP control: 6	Boys: 2 Girls: 4	15.7 ± 1.2	GMFCS levels II and III	Bilateral spastic: 6
		CP intervention: 7	Boys: 2 Girls: 5	15.9 ± 1.5	GMFCS levels II and III	Bilateral spastic: 7
**Van Wely et al.**^52^^,^‡	RCT	CP control: 24	Boys: 16 Girls: 8	10.0 ± 1.8	GMFCS I: 13 GMFCS II: 6 GMFCS III: 5	Unilateral spastic: 11 Bilateral spastic: 13
		CP intervention: 25	Boys: 12 Girls: 13	9.5 ± 1.5	GMFCS I: 15 GMFCS II: 6 GMFCS III: 4	Unilateral spastic: 12 Bilateral spastic: 13
**Verschuren et al.**^53^^,^‡	Clinimetric study (compares 2 test modalities)	CP: 25	Boys: 15 Girls: 10	11.5 ± 2.8 12.5 ± 3.0	GMFCS I: 14 GMFCS II: 11	NR
**Verschuren and Takken**^20^^,^‡	Cross-sectional study	CP: 24	Boys: 16 Girls: 8	11.2 ± 2.8 12.5 ± 3.0	GMFCS I: 13 GMFCS II: 11	Unilateral spastic: 12 Bilateral spastic: 12
**Verschuren et al.**^54^	Clinimetric study (compares 2 test modalities)	CP: 23	Boys: 18 Girls: 5	13.3 ± 3.6	GMFCS III: 3 GMFCS IV: 20	Spastic: 23
**Zwinkels et al.**^55^^,^‡ **2004-matched sample**	Comparing 2004 and 2014 samples	CP: 15	Boys: 10 Girls: 5	11.9 ± 2.8 13.4 ± 2.0	GMFCS I: 8 GMFCS II: 7	Spastic: 15
**2014-matched sample**		CP: 15	Boys: 10 Girls: 5	12.0 ± 2.7 13.5 ± 2.9	GMFCS I: 8 GMFCS II: 7	Spastic: 15
**Total**#		**TD: 180** **CP: 843**	**Boys: 586** **Girls: 398** ***Missing:39***	**CP: 12.62 (SE 0.54)**⁎⁎ **TD: 12.43 (SE 0.72)**⁎⁎	**GMFCS I: 302** **GMFCS II: 242** **GMFCS III: 67** **GMFCS IV: 38** **GMFCS V: 0** ***Missing: 194***	**Unilateral spastic: 245** **Bilateral spastic: 421** **Spastic: 103** **Dyskinetic: 9** **Ataxic: 2** ***Missing: 63***

‡ Study shows overlap in participants with other study/studies.

⁎ Data were available per participant, and therefore the mean and standard deviation were manually calculated.

# Data reported as median (IQR).

⁎⁎ Pooled average of age of unique children Abbreviations: CCT, controlled clinical trial; CP, cerebral palsy; n, number; na, not applicable; NR, not reported; RCT, Randomized Controlled Trial; SD, standard deviation; SE, standard error; TD, typically developing; UCT, uncontrolled clinical trial.

Among the 36 included studies, there was overlap between study populations in 16 studies (44.4 %).^20^, ^21^, ^22^, ^23^, ^24^, ^25^, ^26^, ^27^, ^28^, ^29^, ^30^, ^31^, ^32^, ^33^, ^34^, ^35^, ^36^, ^37^, ^38^, ^39^, ^40^, ^41^, ^42^, ^43^, ^44^, ^45^, ^46^, ^47^, ^48^, ^49^, ^50^, ^51^, ^52^, ^53^, ^54^, ^55^ The study populations of Hoofwijk et al.^32^ and Unnithan et al.^50^ were identical, and therefore only the paper of Hoofwijk et al.^32^ was used in this review. There was overlap in study populations in the papers of Verschuren and Takken,^20^ Verschuren et al.,^53^ and Zwinkels et al.^55^ For pooling of the child characteristics the 2004 cohort data from Verschuren et al.^53^ and the 2014 cohort described by Zwinkels et al.^55^ were used. The papers of Verschuren and Takken^20^ and Verschuren et al.^53^^,^^54^ were used for subgroup meta-analysis. Nsenga Leunkeu et al.^44^^,^^45^ also reported training results of the same children in two articles. Data from the 2013 article^45^ were used for pooling. The trial population of van Wely et al.^52^ was used for secondary analyses by Balemans et al.^24^^,^^25^ In the largest study with 70 children (Balemans et al.^23^), trial participants, but also children from the study by Brehm et al. ,^27^ were included. In this systematic review, the largest study by Balemans et al.^23^ was the starting point for pooling unique VO_2peak_ data. The papers of Kim et al.,^34^ Lee et al..^39^ Park et al.,^46^ and Suk and Kwon^49^ also had an overlap in study populations. In this case, the largest data set of Kim et al.^34^ was used.

### Characteristics of the included participants

Taking the overlap between studies into account, 510 unique children with CP and 173 unique TD peers were included. Table 1 shows the extracted child characteristics (age, sex, GMFCS, and distribution and motor type of CP) of each included paper. Boys, and children with bilateral spastic CP at GMFCS levels I and II were in the majority.

### VO_2peak_ related to child characteristics

VO_2peak_ was measured using bicycle ergometer (*n* = 16), treadmill walking (*n* = 13), arm crank ergometer (*n* = 6), shuttle run test (*n* = 3), and shuttle ride test (*n* = 1). VO_2peak_ values and physiological characteristics per study are presented in Supplementary material Table S3.

**Fig. 2:**
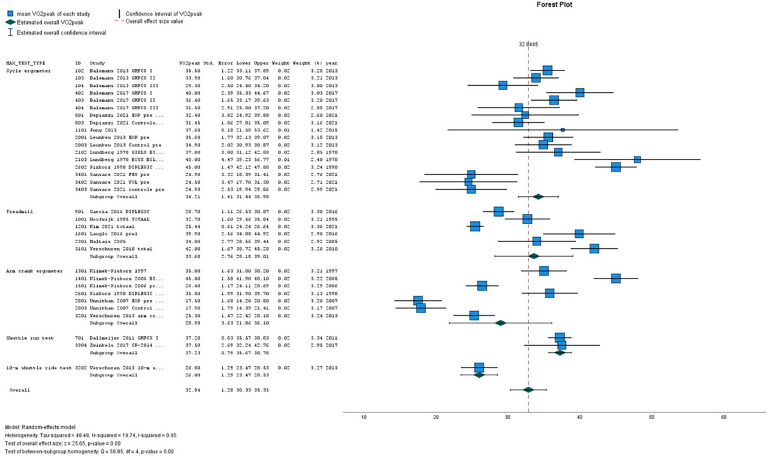
Forest plot of VO_2peak_ outcomes (in mL/kg/min) in children with CP. Studies are ordered by type of exercise test.

**Fig. 3:**
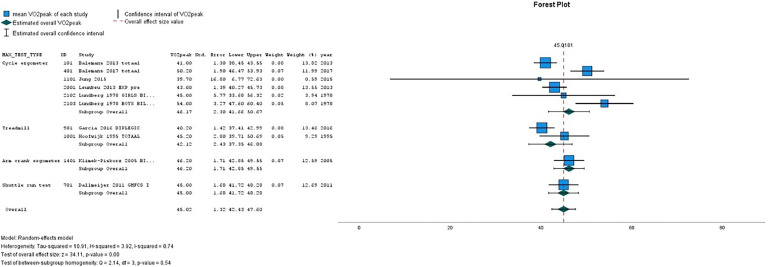
Forest plot of VO2peak outcomes (in mL/kg/min) in TD children. Studies are ordered by type of exercise test.

Table 2(A-D) presents the pooled VO_2peak_ (in mL/kg/min) in subgroups of children with CP and TD peers. The overall estimated VO_2peak_ in children with CP was 32.84 mL/kg/min (SE 1.28) and 45.02 mL/kg/min (SE 1.32) in TD peers, with a mean difference between CP and TD of −12.17 mL/kg/min (95 % CI diff: −16.70, −7.64). (Table 2A) Fig. 2, Fig. 3 show the forest plots of study outcomes in children with CP and TD peers.

**Table 2:** VO_2peak_ estimates in children with cerebral palsy and typically developing children, resulting from meta-analyses.

2A. VO2peak estimates per type of maximal exercise test										
	Children with cerebral palsy					**Typically developing children**				
**Type of exercise test**	Studies n	VO_2peak_ (mL/kg/min)	Std. Error	95 % Confidence Interval		Studies n	VO_2peak_ (mL/kg/min)	Std. Error	95 % Confidence Interval	
				Lower	Upper				Lower	Upper
Cycle ergometer	8	34.21	1.41	31.44	36.98	5	46.17	2.30	41.66	50.67
Treadmill	6	33.60	2.76	28.18	39.01	2	42.12	2.43	37.35	46.88
Arm crank ergometer	6	28.98	3.63	21.86	36.10	1	46.20	1.71	42.85	49.55
Shuttle run test	2	37.23	0.79	35.67	38.78	1	45.00	1.68	41.72	48.29
10-m shuttle ride test	1	26.00	1.29	23.47	28.53	–	–	–	–	–
Overall	22*	32.84	1.28	30.33	35.36	9	45.02	1.32	42.43	47.61
* number of unique studies										

2B VO_2peak_ estimates of boys and girls										
	**Children with cerebral palsy**					**Typically developing children**				
**Sex**	Studies n	VO_2peak_ (mL/kg/min)	Std. Error	95 % Confidence Interval		Studies n	VO_2peak_ (mL/kg/min)	Std. Error	95 % Confidence Interval	
				Lower	Upper				Lower	Upper
Boys	7	39.43	3.50	32.57	46.30	3	48.84	1.99	44.94	52.75
Girls	3	34.64	1.26	32.17	37.11	2	37.74	5.74	26.49	49.00
Overall	6*	38.23	2.58	33.17	43.30	3	45.21	4.38	36.63	53.80
* number of unique studies										

2C. VO_2peak_ estimates per GMFCS level										
**Children with cerebral palsy**										
**GMFCS level**	Studies n	VO_2peak_ (mL/kg/min)	Std. Error	95 % Confidence Interval						
				Lower	Upper					
I	5	35.41	2.94	29.65	41.17					
II	5	32.05	2.57	27.01	37.09					
III	2	30.23	1.90	26.52	33.95					
III/IV	1	25.70	0.97	23.80	27.60					
Overall	7*	32.01	1.55	28.97	35.06					
* number of unique studies										

2D. VO_2peak_ estimates per type of motor distribution										
**Children with cerebral palsy**										
**Motor distribution**	Studies n	VO_2peak_ (mL/kg/min)	Std. Error	95 % Confidence Interval						
				Lower	Upper					
Bilateral	8	32.77	3.15	26.60	38.95					
Unilateral	1	33.50	1.21	31.13	35.87					
Overall	8*	32.81	2.77	27.38	38.23					

⁎ number of unique studies *Abbreviations: GMFCS, Gross Motor Function Classification System; n, number;* VO_2peak_*, peak oxygen uptake*.

On all types of exercise tests, TD peers scored higher VO_2peak_ values than children with CP (Table 2A). In children with CP, the highest pooled VO_2peak_ was found on the shuttle run test: 37.23 mL/kg/min (SE 0.79).

Subgroup meta-analysis of six studies^20^^,^^32^^,^^36^^,^^37^^,^^41^^,^^47^ revealed that boys with CP scored higher VO_2peak_ values than girls with CP: VO_2peak_ of 39.43 mL/kg/min (SE 3.50) and 34.64 mL/kg/min (SE 1.26), respectively, with a mean difference of 4.79 mL/kg/min (95 % CI: −8.10, 17.68). The sex-specific VO_2peak_ data of TD peers were non-significantly higher (Table 2B). The difference between boys with CP and TD boys equalled −9.41 mL/kg/min (95 % CI: −22.47, 3.65); between girls with CP and TD girls −3.10 mL/kg/min (95 % CI: −17.68, 11.47).

Overall, seven studies reported on the VO_2peak_ per GMFCS level, showing gradual differences between children at GMFCS level I, II, III, and III/IV with respectively a pooled VO_2peak_ of 35.41 (SE 2.94), 32.05 (SE 2.57), 30.23 (SE 1.90), and 25.70 (SE 0.97) mL/kg/min (Table 2C).^23^^,^^26^^,^^29^^,^^30^^,^^34^^,^^54^^,^^55^

Seven studies reported on the VO_2peak_ for CP subgroups with bilateral involvement or unilateral involvement (Table 2D).^25^^,^^31^^,^^36^^,^^37^^,^^41^^,^^47^^,^^51^ Children with bilateral involvement had a pooled VO_2peak_ 32.77 mL/kg/min (SE 3.15); one study^25^ presented VO_2peak_ data of children with unilateral involvement, i.e. 33.50 mL/kg/min (SE 1.21).

## Discussion

This study demonstrated that children with CP had a lower VO_2peak_ compared with TD peers, with most compromised values in children at higher GMFCS levels and boys with CP. These findings call for preventive measures supporting a healthy lifestyle and increased participation in physical activities for young people with CP.^56^

Indeed, it cannot be expected that, on average, children with CP are able to reach VO_2peak_ values like TD peers, due to physical and mental differences. The consequences of brain damage on the musculoskeletal and cardiopulmonary system in children with CP could affect their aerobic fitness. A number of research findings are consistent with this, such as the lower muscle mass and the early switch to anaerobic glycolysis, reduced cardiac output and consequently reduced transport of oxygen to muscles, and lower ventilatory efficiency.^57^, ^58^, ^59^, ^60^

Physical deconditioning might be another explanation for the decreased VO_2peak_ in children with CP. Children with CP tend to be 30 % less physically active compared to their TD peers, and are two times more likely to be engaged in sedentary behavior.^61^, ^62^, ^63^ The life expectancy of individuals with CP has improved in recent decades, and an increasing number of children with CP now survive into adulthood. Therefore, understanding the process of aerobic fitness in CP is important to reduce the risks of low aerobic fitness and to prevent long-term effects across the lifespan.^56^^,^^64^, ^65^, ^66^

Different cardiorespiratory exercise tests were used, all with the common goal that children performed until exhaustion and consequently reached VO_2peak_ values. The choice of exercise test is often based on the motor capabilities of children. Children at higher GMFCS levels (III and IV) have more restricted functional mobility and, as a result, mainly participated in arm crank ergometer tests and shuttle ride tests.^54^ The study of Lauglo et al.^38^ showed that children at GMFCS levels III and IV were able to perform a treadmill exercise test with the use of a body weight support system, but VO_2peak_ values were still not reached. In children who are not able to self-propel a manual wheelchair (GMFCS level V), it is not feasible to perform a maximal exercise test to directly measure their VO_2peak_.

When evaluating the relation between VO_2peak_ and the level of functional mobility classified using the GMFCS, it became clear that the VO_2peak_ gradually decreased in children with more mobility limitations. In these children the performed activities are probably quickly supplemented by the anaerobic metabolism, limiting sustained exercising for a long period of time.^67^ The inverse relationship between GMFCS level and physical activity calls for personalized strategies to increase physical activity in children with CP.^54^^,^^63^

The finding of the present meta-analysis that boys scored higher VO_2peak_ values than girls, is consistent with previous studies.^68^, ^69^, ^70^, ^71^ Body composition is an important predictor for VO_2peak_, as boys generally have greater muscle mass and a lower proportion of body fat.^68^^,^^72^ Moreover, the cardiopulmonary system of boys is probably more capable to drive them to maximal levels.^73^ However, according to Dencker et al.^68^ sex differences could not solely be explained by the aforementioned factors, so more research is needed to explore determinants of aerobic fitness in girls and boys.

### Societal and clinical implications

With regard to clinical practice and public health, it is highly relevant to understand the impact of low aerobic fitness of children with CP. A low aerobic fitness in childhood disability has clearly proven to have negative health consequences in the short term as well as the long term. Maximum oxygen uptake values below the threshold of 42 mL/kg/min for boys and 35 mL/kg/min for girls indicate potential cardiovascular risk.^9^ Our meta-analysis showed that both boys and girls with CP scored below these minimal recommended thresholds associated with positive health. Taken together, these findings highlight the importance to identify and monitor children with increased cardiovascular disease risk, and to utilize all opportunities to improve their aerobic fitness at the start of childhood.^6^^,^^7^^,^^9^ Even small increases in cardiorespiratory fitness are associated with considerably lower adverse cardiovascular event rates.^6^

Physical activity is essential for the growth, development, well-being, and socialization of every child, especially children with disabilities.^4^^,^^10^^,^^74^ It is not obvious for every child to pursue a healthy lifestyle and avoid sedentary behavior. Several barriers to being physically active for children with a disability are identified.^74^, ^75^, ^76^, ^77^ Better sporting facilities for children with a disability, and more awareness is needed to keep children and parents informed about available possibilities. To reach this goal, co-creation, teamwork, and intersectoral collaboration remains required between the child, parents, health care professionals (e.g. pediatric physicians and pediatric physical therapists), schools, sport coaches, and municipality.^4^^,^^10^^,^^78^

### Study limitations

To the best of our knowledge, no previous systematic review and meta-analysis has been published summarizing the VO_2peak_ in children with CP compared with TD peers. VO_2peak_ values were measured directly by maximal exercise tests instead of estimated from submaximal tests. However, the results should be viewed in the light of the following limitations. In this systematic review we were not able to use an existing, valid risk of bias tool to evaluate the quality of included studies and weighing the level of evidence according to the GRADE (Grading of Recommendations, Assessment, Development and Evaluation) method.^79^ Considering the main aim of this review, the risk of bias of each study was assessed by means of well-established exercise physiology criteria regarding the unbiased measurement of VO_2peak_.^12^^,^^15^^,^^67^ Based on our quality assessment, some studies reported only data from children who met the VO_2peak_ criteria, while other studies also included children who did not meet the VO_2peak_ criteria in their analyses. In clinical practice, it is quite often difficult for children to comply with the instructions and to reach their maximum exercise level during testing. This may imply that the pooled VO_2peak_ values of children with CP as well as TD children in this review are an underestimation of their maximal aerobic capacity. A difficulty in pooling the data was the large overlap in study populations in 16 of the 36 studies. We carefully analyzed the studies to avoid using duplicate samples. Furthermore, there was a large heterogeneity of included studies, which may be a potential source of bias. For example, the population characteristics of children with CP differed, varying protocols for exercise testing were used, and tests were performed under different conditions (laboratory and field tests).^14^ The current review is based on aggregated data, i.e. combining the grouped (average) data of primary studies published between 1978 and 2023, and reflects the state of pediatric exercise physiology research over the past 50 years. With univariate subgroup analyses we were able to reduce some of the heterogeneity. However, the available aggregated data did not allow further refinement, i.e. multivariable meta-analysis combining different child characteristics.

## Conclusion

This systematic review and meta-analysis showed that the aerobic fitness (i.e. VO_2peak_) in children with CP, as measured by a maximal exercise test, is severely compromised compared with TD peers, indicating that they are at increased cardiovascular risk. In boys with CP compared to TD boys, and children at higher GMFCS levels, aerobic fitness was most compromised. These findings emphasize the importance of increased awareness of monitoring low VO_2peak_ in children with CP and the need to address this in clinical practice as well as in the public health domain. Physical activity and prevention of sedentary behavior are important aspects of a healthy lifestyle to improve aerobic fitness in children with CP. Thus, early integration of physical activities into the daily lives of children with disability, for instance with guided sports and exercise programs in an inclusive society, is necessary to prevent negative health consequences.

## Declaration of competing interest

The authors declare no competing interest.

## Appendix Supplementary materials

Image, application 1
