# Supplementary material for: Aerobic fitness in children with cerebral palsy compared to typically developing peers: A systematic review and meta-analysis
Source: Braz J Phys Ther. 2024 Nov 15;28(6):101142. doi: 10.1016/j.bjpt.2024.101142 (PMC11607667; doi:10.1016/j.bjpt.2024.101142)
Supplement: Supplementary file 1 [file mmc1.pdf]

Supplementary online material

Aerobic fitness in children with cerebral palsy compared with typically developing peers: a systematic review and meta-analysis.

Emma J. Wijnhoud MD, Arnoud M.M. Edelman Bos MSc, Annemieke I. Buizer MD PhD, Heleen Beckerman PhD

S1 Table: Search terms and results

Search completed on November 15, 2023.

Databases		
PubMed (MEDLINE), APA PsycArticles, APA PsycInfo, CINAHL, SPORTDiscus (EBSCO)	Before de-duplicating	After de-duplicating
**Total**	**480**	**398**

PubMed (MEDLINE)

#	Searches	Results
1	"oxygen consumption"[MeSH Terms] OR "oxygen consumption"[Title/Abstract] OR "vo2"[Title/Abstract] OR "aerobic capacity"[Title/Abstract] OR "cardiorespiratory fitness"[Title/Abstract] OR "exercise capacity"[Title/Abstract] OR "oxygen uptake"[Title/Abstract]	164,197
2	"cerebral palsy"[MeSH Terms] OR "cerebral palsy"[Title/Abstract]	32,483
3	"child"[MeSH Terms] OR "adolescent"[MeSH Terms] OR "minors"[MeSH Terms] OR "children"[Title/Abstract] OR "kids"[Title/Abstract] OR "youth"[Title/Abstract] OR "minors"[Title/Abstract] OR "juveniles"[Title/Abstract] OR "boys"[Title/Abstract] OR "girls"[Title/Abstract] OR "preadolescents"[Title/Abstract] OR "teen*"[Title/Abstract] OR "youngsters"[Title/Abstract] OR "adolescents"[Title/Abstract]	3,790,169
**4**	**#1 AND #2 AND #3**	**177**

PsycArticles, PsycInfo, CINAHL & SPORTDiscus (EBCOST)

#	Searches	Results
1	TX aerobic capacity OR TX cardiorespiratory fitness OR TX exercise capacity OR TX oxygen consumption OR TX vo2	67,966
2	TX cerebral palsy	33,810
3	TX children OR TX adolescents OR TX minors OR TX kids OR TX youth OR TX boys OR TX girls OR TX preadolescent OR TX teenagers OR TX teens OR TX youngsters	3,160,994
**4**	**#1 AND #2 AND #3**	**303**

S2 Table: Main characteristics and quality assessment of the 36 included studies, alphabetically ordered by first author per maximal exercise test used.

	Study	Study country	Data used more than once?‡	Data used for overall pooling in meta-analysis?	Children with CP at inclusion (*n*)	TD peers at inclusion (*n*)	Quality assessment (i.e. Risk of Biased VO_{2peak} measurement)		
							Reported how VO_{2peak} was defined?	Prior stated additional peak criteria (e.g. HR, RER, exertion)?	Reporting the number of children who successfully completed the maximal exercise test?
	**Bicycle Ergometer**								
1	Balemans et al. (2013)	Netherlands	Yes	**Yes**	70	**31**	Yes	Yes	Yes
2	Balemans et al. (2015)	Netherlands	Yes	No	46		Yes	Yes	Yes
3	Balemans et al. (2015)	Netherlands	Yes	No	46		Yes	Yes	Yes
4	Balemans et al. (2017)	Netherlands	No	**Yes**	37	**20**	Yes	Yes	Yes
5	Brehm et al. (2014)	Netherlands	Yes	No	21		Yes	Yes	Yes
6	Depiazzi et al. (2021)	Australia	No	**Yes**	12		Yes	Yes	Yes
7	Jung et al. (2015)	Republic of Korea	No	**Yes**	4	**2**	Yes	No	Yes
8	Leunkeu et al. (2005)	France	No	No	9	10	Yes	Yes	No
9	Leunkeu et al. (2009)	France	No	No	8	8	Yes	Yes	No
10	Nsenga Leunkeu et al. (2012)	France	Yes	No	28		Yes	No	Yes
11	Nsenga et al.(2013)	France	Yes	**Yes**	24	**12**	Yes	No	Yes
12	Lundberg (1978)	Sweden	No	**Yes**	9	**9**	No	Yes	Yes
13	Lundberg (1984)	Sweden	No	No	26	12	No	No	Yes
14	Van Wely et al. (2014)	Netherlands	Yes	No	49		No	No	Yes
15	Sansare et al. (2021)	United Stated of America	No	**Yes**	15		Yes	No	Yes
	**Treadmill exercise test**								
16	Dahlbäck and Norlin (1985)	Sweden	No	No	6		No	No	Yes
17	Garcia et al. (2016)	Spain	No	**Yes**	40	**40**	Yes	Yes	Yes
18	Hoofwijk et al. (1995)	Canada and United Kingdom	Yes	**Yes**	9	**9**	Yes	Yes	Yes
19	Kim et al. (2021)	Republic of Korea	Yes	**Yes**	40		No	No	Yes
20	Lauglo et al. (2016)	Norway	No	**Yes**	20		Yes	Yes	Yes
21	Lee et al. (2023)	Republic of Korea	Yes	No	39		No	No	Yes
22	Maltais et al. (2005)	Canada	No	**Yes**	11		Yes	Yes	Yes
23	Massin and Allington (1999)	Belgium	No	No	15		No	No	Yes
24	Park et al. (2021)	Republic of Korea	Yes	No	26		No	No	Yes
25	Unnithan et al.(1996)	Canada	Yes	No	9	9	Yes	No	Yes
26	Verschuren and Takken (2010)	Netherlands	Yes	**Yes**	24		No	Yes	Yes
27	Suk and Kwon (2022)	Republic of Korea	Yes	No	46		No	No	Yes

	**Arm crank ergometer test**								
28	Klimek-Piskorz and Piskorz (1997)	Poland	No	**Yes**	14		No	No	Yes
29	Klimek-Piskorz et al.(2005)	Poland	No	**Yes**	10	**10**	Yes	Yes	Yes
30	Klimek-Piskorz (2006)	Poland	No	**Yes**	8		Yes	No	Yes
31	Unnithan et al.(2007)	Greece	No	**Yes**	14		No	Yes	Yes
	**Shuttle Run Test**								
32	Dallmeijer and Brehm (2011)	Netherlands	No	**Yes**	8	**10**	Yes	Yes	Yes
33	Zwinkels et al. (2017)	Netherlands	Yes	**Partly**	15		No	Yes	Yes
	**Two types of exercise tests**								
34	Verschuren et al.(2006)	Netherlands	Yes	Partly	25		No	Yes	Yes
35	Piskorz and Klimek-Piskorz (1998)	Poland	No	**Yes**	15		Yes	No	Yes
36	Verschuren et al. (2013)	Netherlands	No	**Yes**	23		Yes	Yes	Yes

Abbreviations: CP = cerebral palsy; n = number; VO_{2peak} *= peak oxygen uptake; HR = heart rate; RER = respiratory exchange ratio.*

S3 Table: VO_{2peak} and physiological characteristics of the 36 included studies

Study	Type of maximal exercise test	Children with CP			TD peers		
		VO_{2peak}, *mL/kg/min* (*mean ± SD*) [*range*]	HR_{max}, *bpm* (*mean ± SD*) [*range*]	RER (*mean ± SD*)	VO_{2peak}, *mL/kg/min* (*mean ± SD*)	HR_{max}, *bpm* (*mean ± SD*)	RER (*mean ± SD*)
**Balemans et al. (2013)**	Cycle ergometer test	GMFCS I (n=35): 35.5 ± 7.20*<br>GMFCS II (n=20): 33.9 ± 7.16*<br>GMFCS III (n=8): 29.3 ± 7.07*	GMFCS I: 192 ± 1.7<br>GMFCS II (n=23): 187 ± 2.1<br>GMFCS III: 187 ± 3.6	GMFCS I (n=32): 1.11 ± 0.02<br>GMFCS II (n=17): 1.14 ± 0.02<br>GMFCS III (n=7): 1.13 ± 0.04	41.0 ± 7.24* (n=31)	194 ± 1.8	NR
**Balemans et al. (2015)**	Cycle ergometer test	Overall (n=38): 31.4 ± 6.2<br>Unilateral spastic CP (n=20): 33.5 ± 5.4<br>Bilateral spastic CP (n=18): 29.0 ± 6.3	NR	NR		No TD peers group	
**Balemans et al. (2015)**	Cycle ergometer test	Overall (n=38): 31.4 ± 6.2<br>Unilateral spastic CP (n=20): 33.5 ± 5.4<br>Bilateral spastic CP (n=18): 29.0 ± 6.3	NR	NR		No TD peers group	
**Balemans et al. (2017)**	Cycle ergometer test	GMFCS I (n=13): 40.0 ± 8.6<br>GMFCS II (n=17): 36.4 ± 6.8<br>GMFCS III (n=7): 31.5 ± 7.7	GMFCS I: 190 ± 12<br>GMFCS II: 190 ± 8<br>GMFCS III: 181 ± 11	GMFCS I: 1.09 ± 0.14<br>GMFCS II: 1.12 ± 0.16<br>GMFCS III: 1.07 ± 0.07	50.2 ± 8.5 (n=20)	193 ± 9	1.06 ± 0.05
**Brehm et al. (2014)**	Cycle ergometer test	39.8 ± 7.7 (n=16)	190 ± 11	1.05 ± 0.14		No TD peers group	
**Dahlbäck and Norlin (1985)**	Treadmill exercise test	NR	152 ± 8	0.92 ± 0.06		No TD peers group	
**Dallmeijer and Brehm (2011)**	Shuttle run test	37.2 ± 2.2 (n=7)	197 ± 8 (n=6)	1.02 ± 0.09 (n=7)	45.0 ± 5.3 (n=10)	200 ± 7 (n=10)	0.96 ± 0.07 (n=10)
**Depiazzi et al. (2021)**	Cycle ergometer test	Control group (n=6): 31.45 ± 4.55<br>Intervention group (n=6): 32.40 ± 9.35	Control group: 176 ± 16.8<br>Intervention group: 175 ± 11.2	1.18 (range 1.03 ± 1.24) (n=12)		No TD peers group	
**Garcia et al. (2016)**	Treadmill exercise test	28.7 ± 7.0 (n=40)	NR	NR	40.2 ± 9.0 (n=40)	NR	NR
**Hoofwijk et al. (1995)**	Treadmill exercise test	Overall (n=9): 32.7 ± 4.8<br>Boys (n=7): 32.6 ± 5.4<br>Girls (n=2): 33.3 ± 2.3	Overall: 189 ± 17<br>Boys: 191 ± 19<br>Girls: 183 ± 4	Overall: 1.14 ± 0.06<br>Boys: 1.14 ± 0.06<br>Girls: 1.13 ± 0.08	Overall (n=9): 45.20 ± 8.4<br>Boys (n=7): 48.6 ± 5.7	Overall: 197 ± 9<br>Boys: 195 ± 9<br>Girls: 205 ± 4	Overall: 1.15 ± 0.06<br>Boys: 1.16 ± 0.05<br>Girls: 1.13 ± 0.08

					Girls (n=2): 33.2 ± 1.6		
**Jung et al. (2015)**	Cycle ergometer test	37.6 ± 16.35* (n=4)	138 ± 8.07	1.06 ± 0.18	39.7 ± 23.76* (n=2)	120 ± 15	0.90 ± 0.08
**Kim et al. (2021)**	Treadmill exercise test	Overall (n=37): 25.44 ± 3.73<br>GMFCS I (n=21): 26.20 ± 4.13<br>GMFCS II (n=16): 24.42 ± 2.94	Overall: 167.08 ± 12.75<br>GMFCS I: 165.38 ± 15.07<br>GMFCS II: 169.31 ± 8.83	Overall: 0.98 ± 0.05<br>GMFCS I: 0.97 ± 0.05<br>GMFCS II: 0.99 ± 0.04		No TD peers group	
**Klimek-Piskorz and Piskorz (1997)**	Arm crank ergometer test	35.0 ± 6.1 (n=14)	182 ± 15	NR		No TD peers group	
**Klimek-Piskorz et al. (2005)**	Arm crank ergometer test	45.0 ± 5.0 (n=10)	180.1 ± 7.0	NR	46.2 ± 5.4 (n=10)	199.0 ± 6.5	NR
**Klimek-Piskorz (2006)**	Cycle ergometer test	26.4 ± 3.3 (n=8)	177 ± 12	NR		No TD peers group	
**Lauglo et al. (2016)[#]**	Treadmill exercise test	39.9 (30.5-45.0) (n=8)	193 (186-199)	NR		No TD peers group	
**Lee et al. (2023)**	Treadmill exercise test	25.50 ± 3.58 (n=39)	167.41 ± 13.83	0.98 ± 0.05		No TD peers group	
**Leunkeu et al. (2005)**	Cycle ergometer test	20.6 (n=9)	NR	NR		No average data available	
**Leunkeu et al. (2009)**	Cycle ergometer test	31.27 ± 12.04#	No average data available	NR		No average data available	
**Nsenga Leunkeu et al. (2012)**	Cycle ergometer test	32.3 ± 6.3 (n=28)	148.4 ± 25.1	NR		No average data available	
**Nsenga et al. (2013)**	Cycle ergometer test	Control group (n=10): 34.9 ± 6.4<br>Intervention group (n=10): 35.6 ± 5.6	Control group: 181.1 ± 10.0<br>Intervention group: 181.1 ± 10.2	NR	43.0 ± 4.4 (n=10)	197.2 ± 18.0	NR
**Lundberg (1978)**	Cycle ergometer test	Boys (n=5): 48 ± 10<br>Girls (n=4): 37 ± 6	Boys: 196 ± 5<br>Girls: 195 ± 11	Boys: 0.90 ± 0.09<br>Girls: 0.87 ± 0.04	Boys (n=6): 54 ± 8<br>Girls (n=3): 45 ± 10	Boys: 195 ± 6<br>Girls: 201 ± 8	Boys: 0.96 ± 0.04<br>Girls: 1.06 ± 0.10
**Lundberg (1984)**	Cycle ergometer test	Diplegic boys (n=12):<br>y = 20.9 + 2.00*age<br>Diplegic girls (n=7):<br>y = 31.8 + 0.54*age	Diplegic total (n = 19): y=203.7 – 0.56x	Diplegic total (n = 19): 0.90 ± 0.04<br>Dyskinetic total (n = 4): 0.96 ± 0.03<br>Hemiplegic total (n = 3): 0.99 ± 0.00	Boys (n=7): y = 43.1 + 1.28*age<br>Girls (n=5): y = 47.0 + 0.10*age	Controls total (n = 12): y = 202.1 – 0.55x	Controls total (n = 12): 1.02 ± 0.02
**Maltais et al. (2005)**	Treadmill exercise test	34.0 ± 9.2 (n=11)	189 ± 12	1.05 ± 0.11		No TD peers group	
**Massin and Allington (1999)**	Treadmill exercise test	No average data available	NR	NR		No TD peers group	

**Park et al. (2021)**	Treadmill exercise test	Control group (n=13): 26.62 ± 5.10<br>Intervention group (n=13): 25.25 ± 2.69	Control group: 164.23 ± 14.87<br>Intervention group: 165.62 ± 13.22	NR	No TD peers group		
**Piskorz and Klimek-Piskorz (1998)**	Arm crank ergometer test	35.8 ± 7.7 (n=15)	177 ± 11	NR	No TD peers group		
	Cycle ergometer test	45.0 ± 5.7 (n=15)	179 ± 10	NR	No TD peers group		
**Sansare et al. (2021)**	Cycle ergometer test	Control group (n=11): 24.9 [95%CI: 20-29.9]<br>Intervention group 1 (n=14): 24.9 [95%CI: 18.4-31.4]<br>Intervention group 2 (n=11): 24.5 [95%CI: 17.7-31.4]	Control group: 49 [95%CI: 37-61]<br>Intervention group 1: 61 [95%CI: 45-77]<br>Intervention group 2: 47 [95%CI: 30-64]	NR	No TD peers group		
**Suk and Kwon (2022)**	Treadmill exercise test	Control group (n=23): 26.31<br>Intervention group (n=23): 24.75	Control group: 168.62<br>Intervention group: 169.10	Control group: 0.98<br>Intervention group: 0.98	No TD peers group		
**Unnithan et al. (1996)**	Treadmill exercise test	32.7 ± 4.8 (n=9)	NR	NR	45.2 ± 8.4 (n=9)	NR	NR
**Unnithan et al. (2007)**	Arm crank ergometer test	Control group (n=6): 17.9 ± 4.39<br>Intervention group (n=7): 17.5 ± 4.45	Control group: 152.0 ± 12.5<br>Intervention group: 146.0 ± 20.4	Control group: 1.07 ± 0.13<br>Intervention group: 1.02 ± 0.11	No TD peers group		
**Van Wely et al. (2014)**	Cycle ergometer test	Control group (n=20): 33 ± 6.6<br>Intervention group (n=19): 30 ± 5.3	NR	NR	No TD peers group		
**Verschuren et al. (2006)**	Shuttle run test	NR	GMFCS I: 200.6 ± 6.7<br>GMFCS II: 199.4 ± 6.8	GMFCS I: 1.1 ± 0.1<br>GMFCS II: 1.0 ± 0.1	No TD peers group		
	Treadmill exercise test	n=24<br>GMFCS I: 43.80 ± 6.50<br>GMFCS II: 39.90 ± 9.70	GMFCS I: 192.9 ± 6.2<br>GMFCS II: 193.1 ± 6.1	GMFCS I: 0.9 ± 0.9<br>GMFCS II: 1.0 ± 0.8	No TD peers group		
**Verschuren and Takken (2010)**	Treadmill exercise test	Overall (n=24): 42.0 ± 8.2<br>Boys (n=16): 44.7 ± 7.1<br>Girls (n=8): 36.4 ± 7.6	193.1 ± 6.2	1.0 ± 0.1	External reference data of 336 TD peers were used. These TD peers outcomes were not included in our review		
**Verschuren et al. (2013)**	Arm crank ergometer test	25.3 ± 5.7 (n=23)	160.5 ± 24.5	1.0 ± 0.1	No TD peers group		
	Shuttle ride test	26.0 ± 5.0 (n=23)	171.0 ± 23.2	1.1 ± 0.2	No TD peers group		

**Zwinkels et al. (2017) 2004-matched sample**	Shuttle run test	Overall (n=25): 40.9 ± 8.4<br>GMFCS I (n=15): 41.9 ± 6.2<br>GMFCS II (n=10): 39.7 ± 10.8	198 ± 5	1.05 ± 0.11	No TD peers group
**Zwinkels et al. (2017) 2014-matched sample**	Shuttle run test	Overall (n=25): 37.5 ± 10.4<br>GMFCS I (n=15): 40.2 ± 12.6<br>GMFCS II (n=10): 34.3 ± 5.0	183 ± 18	1.07 ± 0.10	No TD peers group

If reported, the number of participants is noted who completed the test and were included in the analysis.

[*] Data was reported as median (standard error) and is for the purposes of this study converted into median ± SD values.

[#] Data is reported as median (interquartile range).

[‡]Data is reported as ml/kg/lean body mass.

Abbreviations: bpm, beats per minute; CP, cerebral palsy; GMFCS, Gross Motor Function Classification System; n, number; NR, not reported; HR_{max}, maximal heart rate; mL/kg/min, millilitres per kilogram per minute; RER, respiratory exchange ratio; SD, standard deviation; TD, typically developing; VO_{2peak}, peak oxygen uptake.
